# Supplementary material for: Efficient Recovery of Candida auris and Five Other Medically Important Candida Species from Blood Cultures Containing Clinically Relevant Concentrations of Antifungal Agents
Source: Microbiol Spectr. 2023 Jan 30;11(2):e04104-22. doi: 10.1128/spectrum.04104-22 (PMC10100679; doi:10.1128/spectrum.04104-22)
Supplement: Supplemental file 1 — Table S1. Download spectrum.04104-22-s0001.pdf, PDF file, 0.2 MB [file spectrum.04104-22-s0001.pdf]

**TABLE S1** Combinations of *Candida* species and antifungal-drug concentrations for the use in a simulated blood culture model[a]

| **Antifungal drugs**[b] | | | | ***Candida* species**[c] | |
|---|---|---|---|---|---|
| | **Concentration (µg/ml) of antifungal drug to which corresponds a peak serum level of** | | | | |
| **Type** | **100%** | **50%** | **25%** | **Type** | **Minimum inhibitory concentration (µg/ml)**[d] |
| Anidulafungin | 7.2 | 3.6 | 1.8 | *C. auris* | 0.12 |
| | | | | *C. albicans* | 0.015 |
| | | | | *C. glabrata* | 0.06 |
| | | | | *C. krusei* | 0.03 |
| | | | | *C. parapsilosis* | 0.5 |
| | | | | *C. tropicalis* | 0.015 |
| Caspofungin | 9.9 | 5.0 | 2.5 | *C. auris* | 0.25 |
| | | | | *C. albicans* | 0.03 |
| | | | | *C. glabrata* | 0.12 |
| | | | | *C. krusei* | 0.25 |
| | | | | *C. parapsilosis* | 0.5 |
| | | | | *C. tropicalis* | 0.06 |
| Micafungin | 16.4 | 8.2 | 4.1 | *C. auris* | 0.12 |
| | | | | *C. albicans* | 0.015 |
| | | | | *C. glabrata* | 0.03 |
| | | | | *C. krusei* | 0.06 |
| | | | | *C. parapsilosis* | 1 |
| | | | | *C. tropicalis* | 0.03 |
| Fluconazole | 14.0 | 7.0 | 3.5 | *C. albicans* | 0.25 |
| | | | | *C. glabrata* | 8 |
| | | | | *C. parapsilosis* | 1 |
| | | | | *C. tropicalis* | 2 |
| Posaconazole | 3.3 | 1.7 | 0.8 | *C. auris* | 0.25 |
| | | | | *C. albicans* | 0.03 |
| | | | | *C. glabrata* | 0.5 |
| | | | | *C. krusei* | 0.03 |
| | | | | *C. parapsilosis* | 0.03 |
| | | | | *C. tropicalis* | 0.12 |
| Voriconazole | 3.0 | 1.5 | 0.8 | *C. auris* | 2 |
| | | | | *C. albicans* | 0.007 |
| | | | | *C. glabrata* | 0.12 |
| | | | | *C. krusei* | 0.12 |
| | | | | *C. parapsilosis* | 0.03 |
| | | | | *C. tropicalis* | 0.12 |
| Amphotericin B | 3.5 | 1.8 | 0.9 | *C. albicans* | 0.5 |
| | | | | *C. glabrata* | 0.5 |
| | | | | *C. krusei* | 0.5 |
| | | | | *C. parapsilosis* | 0.5 |
| | | | | *C. tropicalis* | 0.5 |

[a]Each antifungal drug's concentration was injected together with one of listed *Candida* species in BACT/ALERT or BACTEC blood culture (BC) bottles, respectively, to simulate BCs of patients under antifungal drug treatment. Test (with antifungal) bottles were compared to control (without antifungal) bottles to assess the growth of *Candida* species in BACT/ALERT VIRTUO or BACTEC FX BC automated instruments.
[b]Antifungal drugs include three echinocandins (anidulafungin, caspofungin, and micafungin), three azoles (fluconazole, posaconazole, and voriconazole), and one polyene (amphotericin B). For each antifungal drug, concentrations were chosen to reflect the relative peak serum level concentrations.
[c]*Candida* species include one clinical isolate (*C. auris* FPG1) and five type/reference strains (*C. albicans* ATCC 90028, *C. glabrata* ATCC 2001, *C. krusei* ATCC 6258, *C. parapsilosis* ATCC 22019, and *C. tropicalis* ATCC 750). The *C. auris* FPG1 designation refers to as the *C. auris* isolate one at the FPG (Fondazione Policlinico Universitario A. Gemelli IRCCS) hospital of Rome (Italy), which is our study's location.
[d]As appropriate, antifungal clinical breakpoints or epidemiological cutoff values (ECVs), established by the Clinical Laboratory Standards Institute (CLSI) respectively in M60 (https://clsi.org/media/1895/m60ed1_sample.pdf) and M57S (https://clsi.org/media/tbvf5qr2/m57sed4e_sample.pdf) documents, were used to interpret minimum inhibitory concentration (MIC) values for five of listed *Candida* species. For the remaining *C. auris*, any MIC to azoles of ≥4 µg/ml (https://doi.org/10.1016/j.cmi.2019.03.028) and a MIC of ≥2 µg/ml to amphotericin B (https://doi.org/10.1128/AAC.00485-17) were, respectively, considered resistant. Only for *C. auris* and *C. krusei*, based on a MIC value showing that the strain/isolate was resistant or intrinsically resistant to the (indicated) antifungal drug, combinations of fluconazole/*C. auris* (MIC, >256 µg/ml), amphotericin B/*C. auris* (MIC, 4 µg/ml), and fluconazole/*C. krusei* (MIC, 32 µg/ml) were excluded from the study.
